# Supplementary figures and images for: Development of a histopathology scoring system for the pulmonary complications of organophosphorus insecticide poisoning in a pig model
Source: PLoS One. 2020 Oct 14;15(10):e0240563. doi: 10.1371/journal.pone.0240563 (PMC7556475; doi:10.1371/journal.pone.0240563)

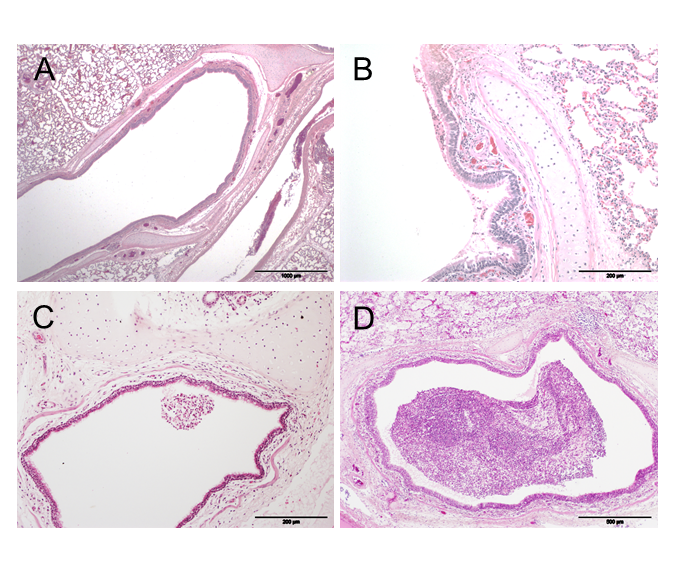

Supplement: S1 Fig — A-D. Number of neutrophils in the bronchial lumens. None (score 0 points; Figure A), <10 per airway (score 1 point; Figure B), 11–50 per airway (score 2 points; Figure C), >50 per airway (score 3 points; Figure D). (TIF) [file pone.0240563.s001.tif]

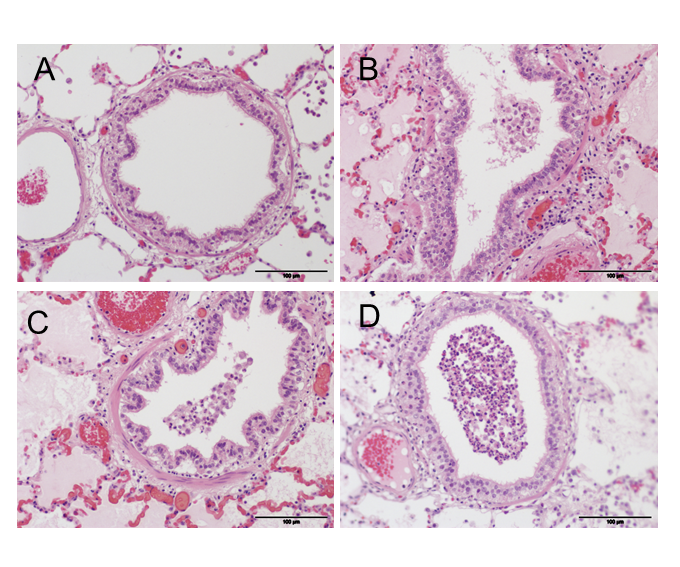

Supplement: S2 Fig — A-D Number of neutrophils in the bronchiolar lumens. None (score 0 points; Figure A), <10 per airway (score 1 point; Figure B), 11–50 per airway (score 2 points; Figure C), >50 per airway (score 3 points; Figure D). (TIF) [file pone.0240563.s002.tif]

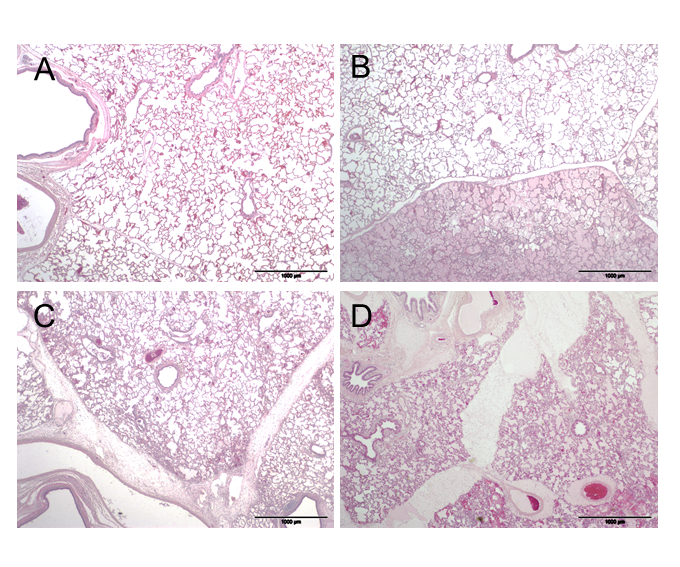

Supplement: S3 Fig — A-D. Presence of edema in the alveoli/interstitium. None (score 0 points; Figure A), <25% (score 1 point; Figure B), 25–50% (score 2 points; Figure C), >50% (score 3 points; Figure D). (TIF) [file pone.0240563.s003.tif]

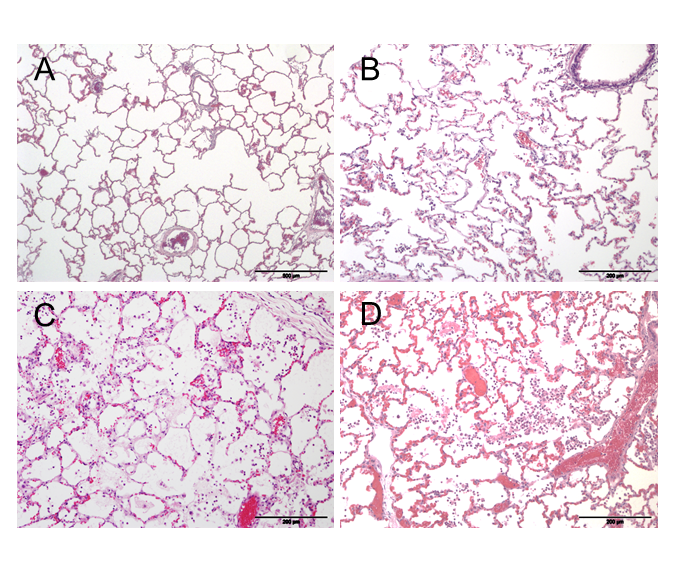

Supplement: S4 Fig — A-D. Numbers of inflammatory cells (mainly neutrophils) in the alveoli. None-few (score 0 points; Figure A), mild increase (score 1 point; Figure B), moderate (score 2 points; Figure C), marked (score 3 points; Figure D). (TIF) [file pone.0240563.s004.tif]

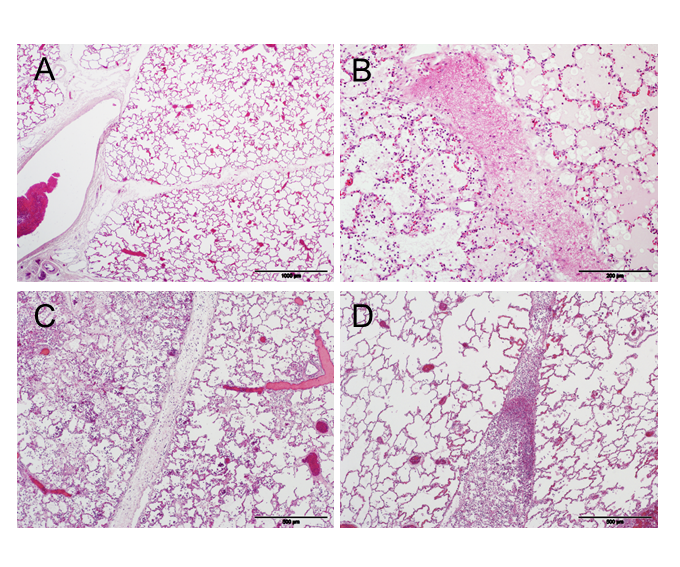

Supplement: S5 Fig — A-D. Numbers of inflammatory cells (mainly neutrophils) in the interstitium. None-few (score 0 points; Figure A), mild increase (score 1 point; Figure B), moderate (score 2 points; Figure C), marked (score 3 points; Figure D). (TIF) [file pone.0240563.s005.tif]

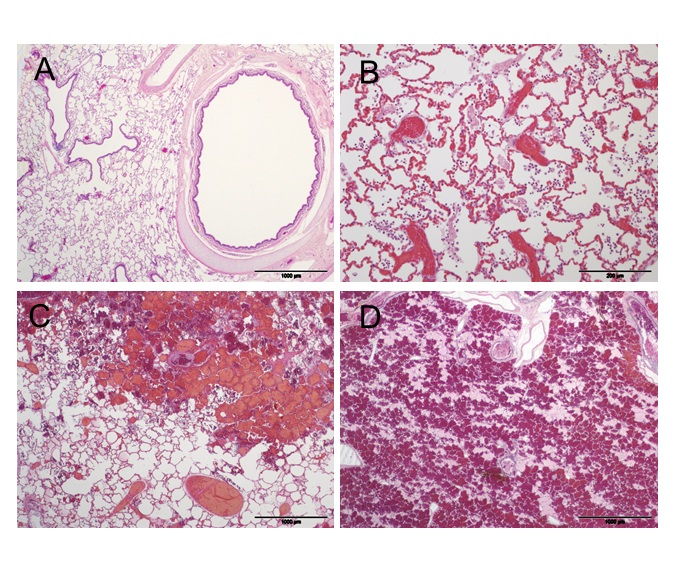

Supplement: S6 Fig — A-D. Presence of hemorrhage/necrosis/fibrin anywhere in sample. None (score 0 points; Figure A), up to 5% (score 1point; Figure B), 5–50% (score 2 points; Figure C), >50% (score 3 points; Figure D). (TIF) [file pone.0240563.s006.tif]
