# Supplementary material for: Development of a histopathology scoring system for the pulmonary complications of organophosphorus insecticide poisoning in a pig model
Source: PLoS One. 2020 Oct 14;15(10):e0240563. doi: 10.1371/journal.pone.0240563 (PMC7556475; doi:10.1371/journal.pone.0240563)
Supplement: Attachment — Submitted filename: Reviewers comments PLOS One PONE D 20-22461.docx [file pone.0240563.s009.docx]

**Reviewer’s comments:**

PONE D-20-22461:

Development and validation of a histopathology scoring system for the pulmonary

complications of organophosphorus insecticide poisoning in a pig model

**General comment:**

I would like to congratulate the authors on compiling this contemporary study. As we all know respiratory failure following organophosphorus pesticides (OP) is very common and is the leading cause of death following self-ingestion of such compounds. I found the article interesting and very easy to follow.

However, for it to be accepted and published, I suggest that the following changes should be made to improve the quality of the article.

**Comment 1:** In line 70 please add a semicolon after NB.

After that I suggest that you add a sentence on the percentages of carbamate, kerosene etc that were found in the solvent the OP compound was dissolved-in to get an idea about the dose response. That way the reader will know that the lung injury was caused by the OP but not the compounds found in the solvent.

**Comment 2:**

The development of the histopathological scoring system is commendable.

**Comment 3:**

In the **Methods** section under the sub sections “ Inter-observer correlation” (line 209) and “Intra-observer correlation” (line 228) , I suggest that you compare the agreement between the different histopathologists and between the same histopathologist by means of Cohen’s kappa statistics and provide a kappa value to quantify the agreement. I believe Krippendorrf’s alpha is a better measurement to compare ordinal values and will give a better agreement. However, even kappa stats can be used for this purpose because it can be done easily in any statistical package that you use. This will give more depth to the message that you all are trying to convey, rather than just stating it is well correlated only. It will solidify the validity of the scoring system in terms of reproducibility.

**Comment 4:**

Is there any specific reason why you selected only caudal and cranial tissue samples from the lung?

**Comment 5:**

Discussion is well written. I suggest that you add a few more sentences to elaborate the applicability of this scoring system to human lung tissues based on the actually existing similarities that you see between human lungs and minipig lungs.
